# *HIF1A*, *EPAS1*, and *VEGFA*: angiogenesis and hypoxia-related gene expression in endometrium and endometrial epithelial tumors

**DOI:** 10.1007/s13353-025-00939-7

**Published:** 2025-01-31

**Authors:** Monika Englert-Golon, Małgorzata Tokłowicz, Aleksandra Żbikowska, Stefan Sajdak, Małgorzata Kotwicka, Paweł Jagodziński, Andrzej Pławski, Mirosław Andrusiewicz

**Affiliations:** 1 Department of Gynecology, Division of Gynecologic Oncology, Poznan University of Medical Sciences, Polna 33 St., 60-535 Poznań, Poland; 2 Department of Cell Biology, Poznan University of Medical Sciences, Rokietnicka 5D, 60-806 Poznań, Poland; 3 Department of Biochemistry and Molecular Biology, Poznan University of Medical Sciences, Święcickiego 6 St., 61-701 Poznań, Poland; 4 Institute of Human Genetics, Polish Academy of Sciences, Strzeszyńska 32 St., 60-479 Poznań, Poland; 5 Collegium Medicum University of Zielona Góra, Zyty 28, 65-046 Zielona Góra, Poland; 6 Poznan University of Medical Sciences, Fredry 10, 61-701 Poznań, Poland

**Keywords:** Endometrial cancer (EC), Hypoxia-inducible factors (HIFs), Hypoxia-inducible factor 1A gene (*HIF1A*), Hypoxia-inducible factor 2A/endothelial PAS domain protein 1 gene (*HIF2A/EPAS1*), Vascular endothelial growth factor A gene (*VEGFA*)

## Abstract

Endometrial cancer (EC) is the second most frequent gynecological malignancy and the sixth most common women’s cancer worldwide. EC incidence rate is increasing rapidly. Apart from the classical, we should consider angiogenesis and hypoxia-related genes as a reason for EC manifestation and progression. We compared the patterns of *HIF1A*, *EPAS1*, and *VEGFA* (genes of interest – GOIs) mRNA expression in 92 cases. *HIF1A* and *VEGFA* levels were higher in EC patients than in controls. *VEGFA* differed significantly between controls and both tumor grades G2 and G3, and we observed a positive correlation for *HIF1A* and *VEGFA* with EC grading. *VEGFA* levels were significantly higher in post-menopausal compared to pre-menopausal patients. All GOIs demonstrated strong correlations in pre-menopausal cases and weak correlations in post-menopausal cases. A positive correlation was observed in pre-menopausal controls for all GOIs and in post-menopausal patients for only *EPAS1* and *VEGFA*. *HIF1A* and *EPAS1* positively correlated with *VEGFA* in post-menopausal EC cases. Multiple linear regression analyses revealed that menopause, body mass index (BMI), and *HIF1A* expression are significant stimulating factors for EC occurrence. *HIF1A* levels were higher in EC patients after BMI and comorbidity number adjustment. The gene-to-gene relation could be seen as either a diagnostic or a therapeutic target in EC. Physicians should inform patients about modifiable risk factors such as BMI. Second, more attention should be paid to diagnosing patients with comorbidities in older age and after menopause. These factors should be considered in designing angiogenesis and hypoxia-related gene-targeting therapies.

**Supplementary Information:**

The online version contains supplementary material available at 10.1007/s13353-025-00939-7.

## Introduction

Endometrial cancer (EC) is the second most frequent gynecological malignancy and the sixth most common women’s cancer worldwide. The Globocan reported 417,367 new cases and 97,370 deaths in 2020. It is Poland’s most common gynecologic malignancy, accounting for 9.7% of cancers in women. In 2020, an estimated 9869 women were diagnosed with EC, and approximately 2195 died (https://gco.iarc.fr/today/home). Moreover, high-income countries have a greater incidence of EC (4.7%) than low-resource countries (4.5%). The International Agency for Research on Cancer reported that the incidence rate of EC is increasing rapidly and is estimated to escalate by more than 50% worldwide by 2040 (Zhang et al. 2019).

ECs have traditionally been classified into type I (grade 1 and 2 endometrioid carcinomas) and type II (grade 3 and non-endometrioid carcinomas). Type I arises from complex atypical hyperplasia and is linked to excessive estrogen stimulation and obesity; patients usually have a good prognosis in the early stages. Type II tumors are less hormone-sensitive and develop from atrophic endometrium with poorly differentiated cells; patients typically show advanced stages with poor prognoses. Type I makes up 70–80% of cases, whereas type II accounts for 10–20% of cases. However, some ECs may present vast heterogeneity, resulting in inadequate histologic characterization. The Proactive Molecular Risk Classifier for Endometrial Cancer (ProMisE) studies revealed that ECs might differ in molecular background. Endometrioid and non-endometrioid cancers have different molecular pathways involving independent sets of genes. ProMisE identified four molecular subgroups characterized by the DNA polymerase *epsilon* (POLE) mutation, mismatch repair deficiency, *TP53* mutation, and a group with no specific molecular profile (Levine et al. 2013). The World Health Organization (WHO) also proposed a new classification of ECs, which includes more detailed information, such as novel immunohistochemical markers, prognostic factors, and recent molecular profiling. According to the up-to-date WHO classification, the histopathologic types of endometrial epithelial tumors are endometrioid carcinoma, mucinous adenocarcinoma, serous adenocarcinoma, clear cell adenocarcinoma, undifferentiated carcinoma, neuroendocrine tumors, and mixed carcinoma composed of more than one type with at least 10% of each component (Englert-Golon et al. 2016; Akhtar et al. 2019; Koskas et al. 2021; Masood and Singh 2021).

The systematic reviews and meta-analyses evaluation revealed that 53 potential risk factors are associated with EC occurrence and mortality. Among them, body mass index (BMI), waist-to-hip ratio, and changes in the level of circulating estrogens in post-menopausal women are strongly related to EC. Moreover, there was a strong association between increasing BMI and both EC types, endometrioid and serous. Other risk factors include reproductive factors such as parity, oral contraceptive use, cigarette smoking, age at menarche, and diabetes, which were associated with both histological groups to similar extents (Reeves et al. 2007; Samulak et al. 2011; Ali 2014; Grandi et al. 2019; Raglan et al. 2019).

Current diagnosis and treatment recommendations for endometrial epithelial tumors are based on clinical examination factors, age, International Federation of Gynecology and Obstetrics (FIGO) stage, histopathologic type and grade, myometrial invasion, and presence of lymph-vascular space invasion. The most recent guidelines have also included the molecular classification for the management of patients with endometrial carcinoma (Concin et al. 2021). As WHO indicated, molecular profiling may be the best way to classify the EC types and provide targeted treatment. While traditional histopathological examination, classification, and grading supported by immunochemistry markers evaluation remain important for diagnosis (Wortman et al. 2019; Masood and Singh 2021), the Polish Society of Gynecological Oncology, based on the current evidence, strongly encourage toward molecular classification of endometrial cancer as the standard of diagnosis (Sznurkowski et al. 2023). The guidelines strongly recommend implementing at least the ProMisE molecular classifier or a more extensive molecular profiling test at the initial diagnosis whenever possible. If immediate molecular classification is infeasible, it should be performed, before making adjuvant treatment decisions. As reviewed by Sznurkowski et al., the rationale for this is that molecular classification provides a more accurate prediction of prognosis and response to therapy than traditional methods (Sznurkowski et al. 2023).

Molecular profiling may also be valuable regarding genes involved in the process of hypoxia. Recent studies have proven that cancer cells present various resistance mechanisms in anticancer therapies. Reduced oxygen availability may regulate the tumor microenvironment and lead to a more aggressive and metastatic phenotype (Wilczak et al. 2010; Jing et al. 2019). On the other hand, the hypoxic tumor microenvironment creates a more favorable niche for infections, which in turn exacerbates the effects of hypoxia, potentially leading to increased tumor aggression and poorer patient outcomes (Udayasuryan et al. 2024). Hypoxia-inducible factors (HIFs) are key regulators in the adaptation of cells, tissues, and organs under hypoxic conditions. In the meta-analysis study, *HIF1A* overexpression was confirmed to be associated with susceptibility, progression, and poor prognosis in the case of EC patients (Zhu et al. 2020). *HIF2A*, also known as endothelial PAS domain protein 1 (*EPAS1*), was also described as a factor related to tumor progression. However, little is known about its role in EC (Luo et al. 2018). *HIF1A* and *HIF2A* regulate a wide range of genes during hypoxia. They may also overlap in activating some target genes, e.g., vascular endothelial growth factor (VEGF), which induces tumor angiogenesis (Wierzbicki et al. 2019).

To the best of our knowledge, there are no studies evaluating the above-described genes in relation to EC in one cohort of patients. Thus, our study aimed to analyze *HIF1A*, *EPAS1*, and *VEGFA* expression patterns in EC tissues and compare them to normal endometrium.

## Materials and methods

### Tissue specimens

The study was conducted according to the guidelines of the Declaration of Helsinki and approved by the IRB of Poznan University of Medical Sciences (PUMS protocol code Nos. 593/19 and 594/19, and the date of approval was 6/19/2019). Written informed consent was obtained from all study participants.

**Fig. 1:**
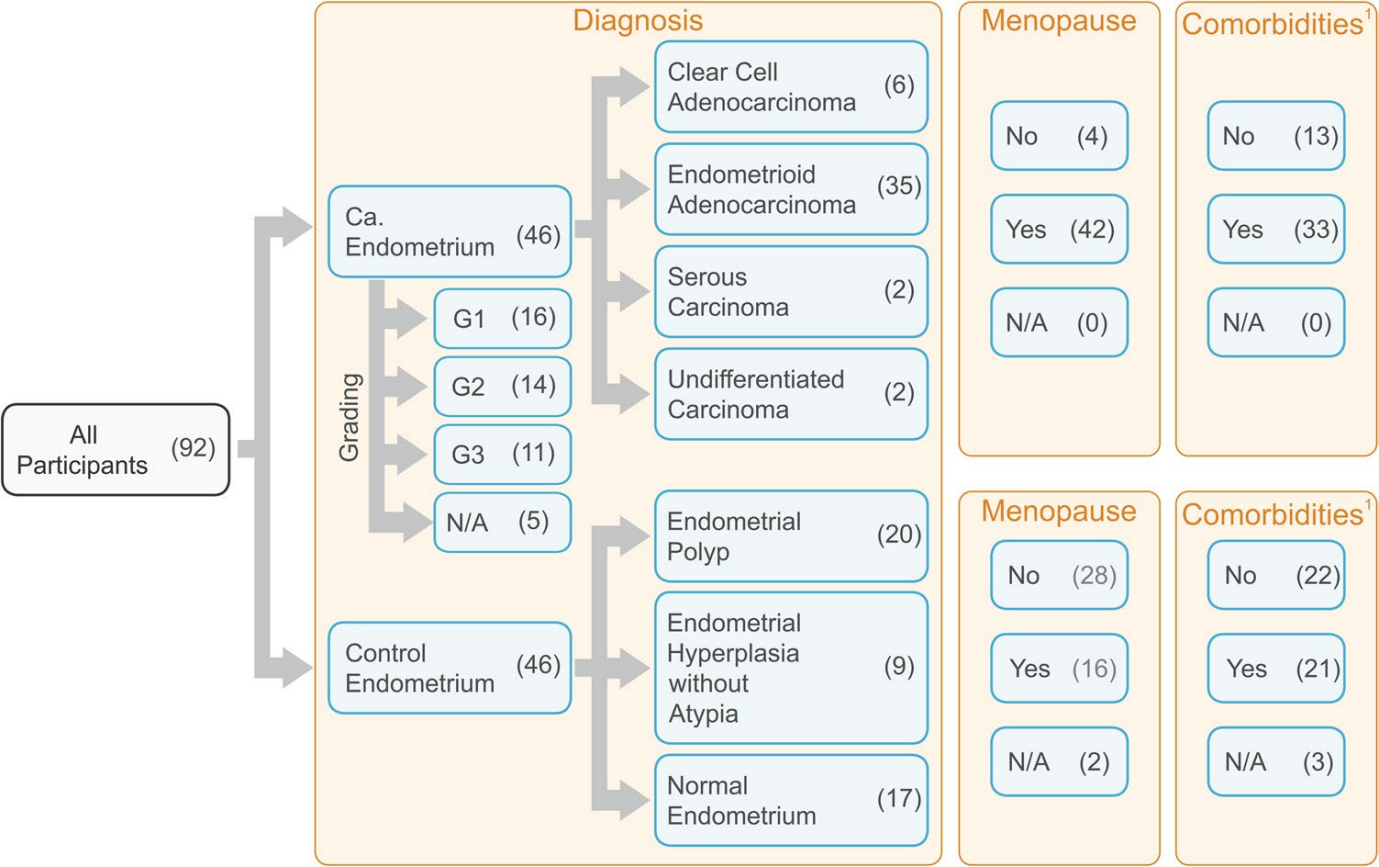
Patients’ characteristics (case number is shown in brackets). N/A, data not available ^1^ — hypertension, diabetes, thyroid lesions, varicose veins, stroke, heart diseases, cataract, glaucoma, COPD, psoriasis, cities, ulcerative colitis, asthma

Between July 2019 and December 2021, 92 women underwent surgery at the Surgical Gynecology Clinic of the Gynecological and Obstetrics Clinical Hospital Poznan University of Medical Sciences. All women were of Caucasian descent. The study cohort consists of tissue specimens obtained from patients with diagnosed endometrial epithelial tumors (*n* = 46). The patients described in our study had not received any cancer-related pharmacological treatment prior to the surgery. Before the surgical procedure, only an endometrial biopsy was performed for diagnosis. Normal, control tissue samples that lack cancerous changes (*n* = 46) were obtained from women who underwent a total hysterectomy. The controls were assessed as a glandular polyp, hypertrophy without atypia, and normal endometrium. The patients’ characteristic is shown in Fig. 1. Anatomicopathological macroscopic and intraoperative microscopic examinations confirmed the absence or presence of cancerous changes. Tissue samples were immersed in an RNA-protective medium (Camacho-Sanchez et al. 2013) and processed at the Chair and Department of Cell Biology, PUMS, or stored at − 80 °C until RNA isolation could be performed.

## Methods

Expression levels of *HIF1A*, *HIF2A*/*EPAS1*, and *VEGFA* were analyzed in different groups. First, we compared controls to EC cases. Gene expression for the genes of interest (GOIs) was also studied in subgroups related to menopausal status, presence of comorbidities (regardless of the cancer manifestation), and grading (G1, G2, and G3).

### Nucleic acid extraction and validation

The microRNA fraction was separated from the total RNA according to the manufacturer’s protocol (cat. no. 035–25; A&A Biotechnology, Gdynia, Poland). High molecular weight RNA’s quality, quantity, and purity were evaluated spectrophotometrically (NanoPhotometer NP-80; IMPLEN, München, Germany). RNA integrity was assessed by electrophoretic separation in denaturing conditions (Andrusiewicz et al. 2016).

### Reverse transcription and quantitative PCR

Complementary to the RNA, DNA was synthesized following the Transcriptor Reverse Transcriptase manufacturer’s protocol (cat. no. 03 531 287,001, Product Information Sheet version 14. Roche Diagnostics GmBH, Basel, Switzerland) in a total volume of 20 μL. The relative expression levels of analyzed GOIs were established using the LightCycler 2.0 carousel glass capillary-based system (Roche, Manheim, Germany) (Andrusiewicz et al. 2016; Englert-Golon et al. 2021). The NCBI accession numbers, probes, and primers’ sequences are described in Table 1. Primer sequences and TaqMan hydrolysis probe positions for the GOIs were determined using the Universal ProbeLibrary (UPL) Assay Design Center algorithm (http://qpcr.probefinder.com, accessed on September 28, 2017).

**Table 1:** The description and location of probes and primers (with sequences) for the self-designed assays

	Reference sequences^%^	TaqMan probe^*&*^	Primers’ sequence (5′ →3′)	Amplicon length [bp]
*HIF1A*^†^	NM_001530.4 NM_001243084.2	No. 71; cat. no. 04688945001	F: tttttcaagcagtaggaattgga^‡^ R: ttccaagaaagtgatgtagtagctg	76
*EPAS1*/*HIF2A*	NM_001430.5	No. 39^‡^; cat. no. 04687973001	F: gaaaacatcagcaagttcatgg R: cagggatgagtgaagtcaaagata	77
*VEGFA*^$^	NM_001025366.3 NM_003376.6 NM_001025367.3 NM_001025368.3 NM_001287044.2	No. 69; cat. no. 04688686001	F: cgaacgtacttgcagatgtga^‡^ R: gagagatctggttcccgaaa	88

*HPRT* assay^&^cat. no. 05532957001; internal control

^%^NCBI GenBank (https://www.ncbi.nlm.nih.gov/genbank/, accessed on September 28, 2017); ^†^transcript variants 1 and 3; ^$^transcript variants 1 – 4 and 10; ^‡^intron spanning ^&^commercially available (Roche, Basel, Switzerland) (Andrusiewicz et al. 2016; Englert-Golon et al. 2021); *bp*, base pairs; *F*, *R*, forward and reverse

Quantitative polymerase chain reactions’ cycling and acquisition steps, standardized for Roche UPL hydrolyzing probes in a total volume of 20 μL, were applied (Andrusiewicz et al. 2016; Skibińska et al. 2018; Janusz et al. 2021; Englert-Golon et al. 2021). Each reaction was performed in duplicate, and the mean values were used for statistical analyses. Reaction efficiencies were obtained from standard curves (Andrusiewicz et al. 2016). Threshold values raw data were analyzed by comparing them to reference curves and *HPRT* reference, using LC 5.0.0.38 software to get concentration ratio values (Cr).

### Statistical analyses

Statistical analyses were performed using Statistica® Version 13.5.0 software for Windows (TIBCO Software Inc., Palo Alto, CA) and PQStat 1.8.0.414 software (PQStat software; Poznan, Poland). All continuous variables were examined for Tukey’s fences for outliers and were winsorized if any were present (Field 2017). The GOIs concentration ratio values were rescaled for each gene separately, using the min–max normalization, and expressed as Cr normalized (Cr norm) values.

Shapiro–Wilk’s test was used to assess data normality. As the data showed non-parametric distribution, two-sided Mann–Whitney *U* and Kruskal–Wallis tests were used with Dunn’s post hoc. A Bonferroni-Hochberg correction was used to test multiple comparisons. Jonckheere-Terpstra trend test was applied to determine the trend in GOI expression level depending on tumor grading as an ordinal variable. To describe experimental results, depending on the distribution of variables, the median [lower–upper quartiles] (Me [Q1-Q3], or mean ± standard deviation (M ± SD) were used. *χ*^2^-test according to Cochran’s rules was applied for nominal data. The odds ratio and 95% confidence intervals (OR, 95% CI) were calculated for the normalized expression level of the genes of interest in the case of controls and malignant tissue specimens. Spearman’s rank correlation tests determined the correlation coefficient (*R*) between parameters. Multiple linear and logistic regression models were used to examine the association of cancer absence or manifestation in analyzed cases. We also analyzed genes’ expression levels adjusted to age, BMI, and comorbidities number. Data were considered statistically significant at *p* < 0.05.

## Results

### Case–control study results

**Fig. 2:**
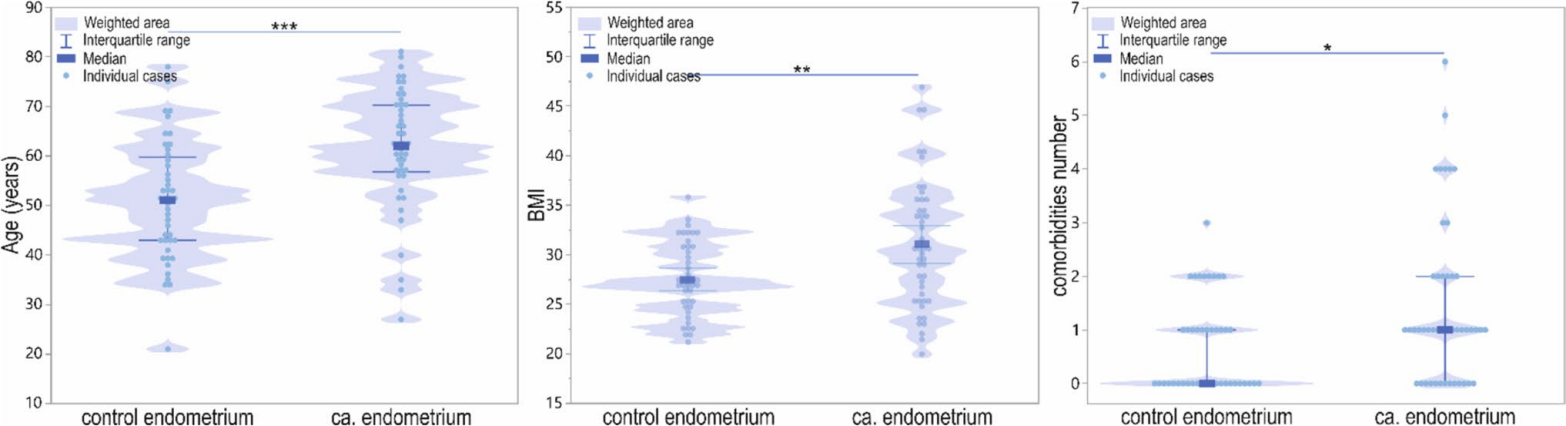
Violin-plot of age, BMI, and comorbidities number in controls and patients; *p < 0.05; **p < 0.01; and ***p < 0.001

There was a significant difference between the control and cancer patient groups in the age (*p* < 0.001), BMI (*p* = 0.002), and the number of comorbidities (*p* = 0.02). The EC patients compared to controls were in more advanced age (Me [Q1–Q3] = 62 [27–81] vs*.* 51 [21–78]), had higher BMI values (M ± SD = 31.04 ± 6.47 vs*.* 27.51 ± 3.70), and manifested a higher number of comorbidities (1 [0–2] maximum comorbidities number 6 vs*.* 1 [0–1] maximum 3; Fig. 2).

We also analyzed the relationship of gene expression with age, BMI, and comorbidity number. *HIF1A* expression did not correlate significantly with the aforementioned parameters (*p* > 0.05). *EPAS1* had a significant positive correlation, yet weak, with age in all analyzed cases (*R* = 0.21, *p* = 0.0492). Regarding the groups, this significant correlation persisted in controls (*R* = 0.33, *p* = 0.0269) but not in cancer patients (*p* > 0.05). There were no significant correlations of *EPAS1* with BMI and comorbidity number. *VEGFA* had a positive, weak, and significant correlation in all cases with age and BMI (*R* = 0.25; *p* = 0.0161, *R* = 0.22; *p* = 0.0389, respectively). The age, BMI, and number of comorbidities did not correlate significantly with *VEGFA* expression in both the control and EC groups (Supplementary Table S1).

**Fig. 3:**
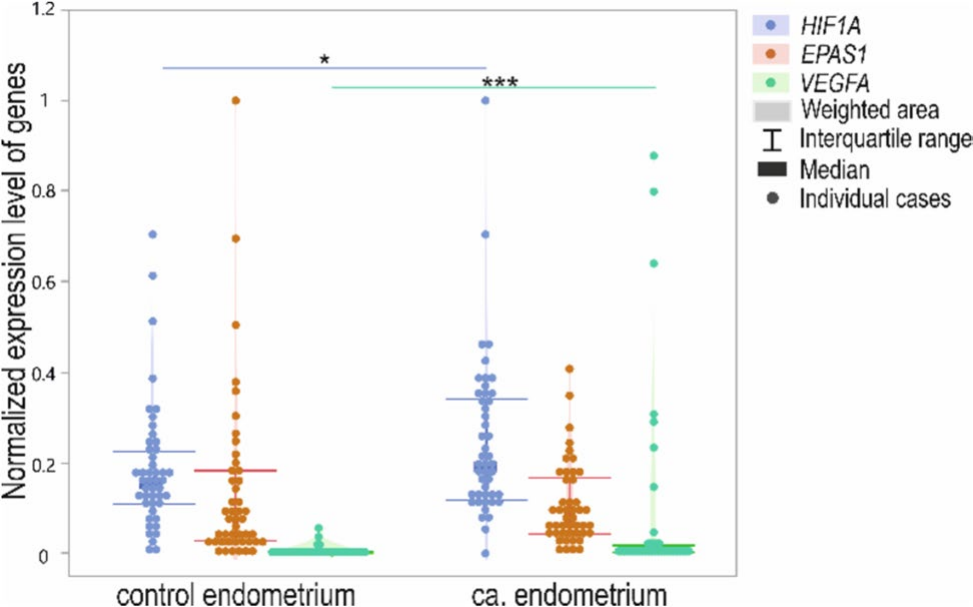
Violin-plot of *HIF1A*, *EPAS1*, and *VEGFA* normalized expression level in controls and patients; *p < 0.05 and ***p < 0.001

The normalized expression level of *HIF1A* and *VEGFA* was significantly higher in endometrial epithelial tumors than in the control group (*p* = 0.048; *p* < 0.001, respectively). There was no difference in *EPAS1* expression levels between the two groups (*p* > 0.05). The min–max normalized expression is presented in Fig. 3. The qPCR expression results of analyzed genes state in line with the data deposited in TCGA database (http://ualcan.path.uab.edu/).

**Fig. 4:**
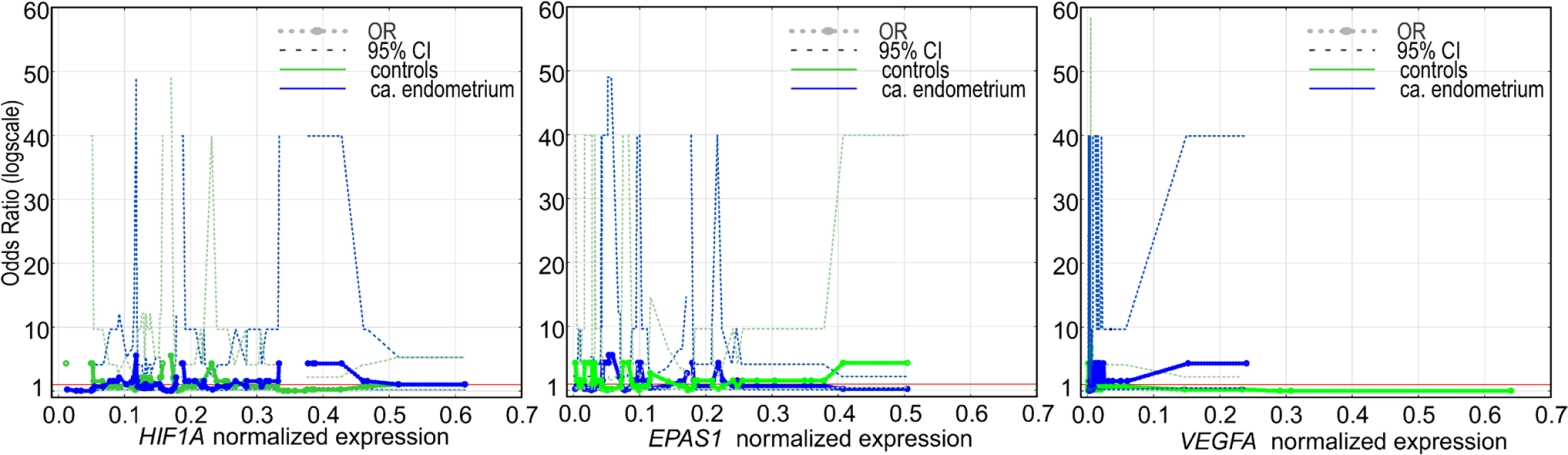
Odds ratio (OR) profiles with 95% confidence intervals (95% CI) for analyzed genes’ expression profiles in controls and cancer-affected specimens. The horizontal line represents OR = 1

Additionally, risk profiles measured by odds ratios with 95% confidence intervals showed in all analyzed genes of interest in most cases that, the expression levels were associated with the susceptibility to tumor manifestation. The expression at a different level was related to promoting or protecting agents in controls and cancer-affected tissues (Fig. 4). The higher expression of *HIF1A* and *EPAS1* was observed in non-affected tissue, whereas *VEGFA*’s slightly higher expression seems to predispose to malignant transformation.

**Fig. 5:**
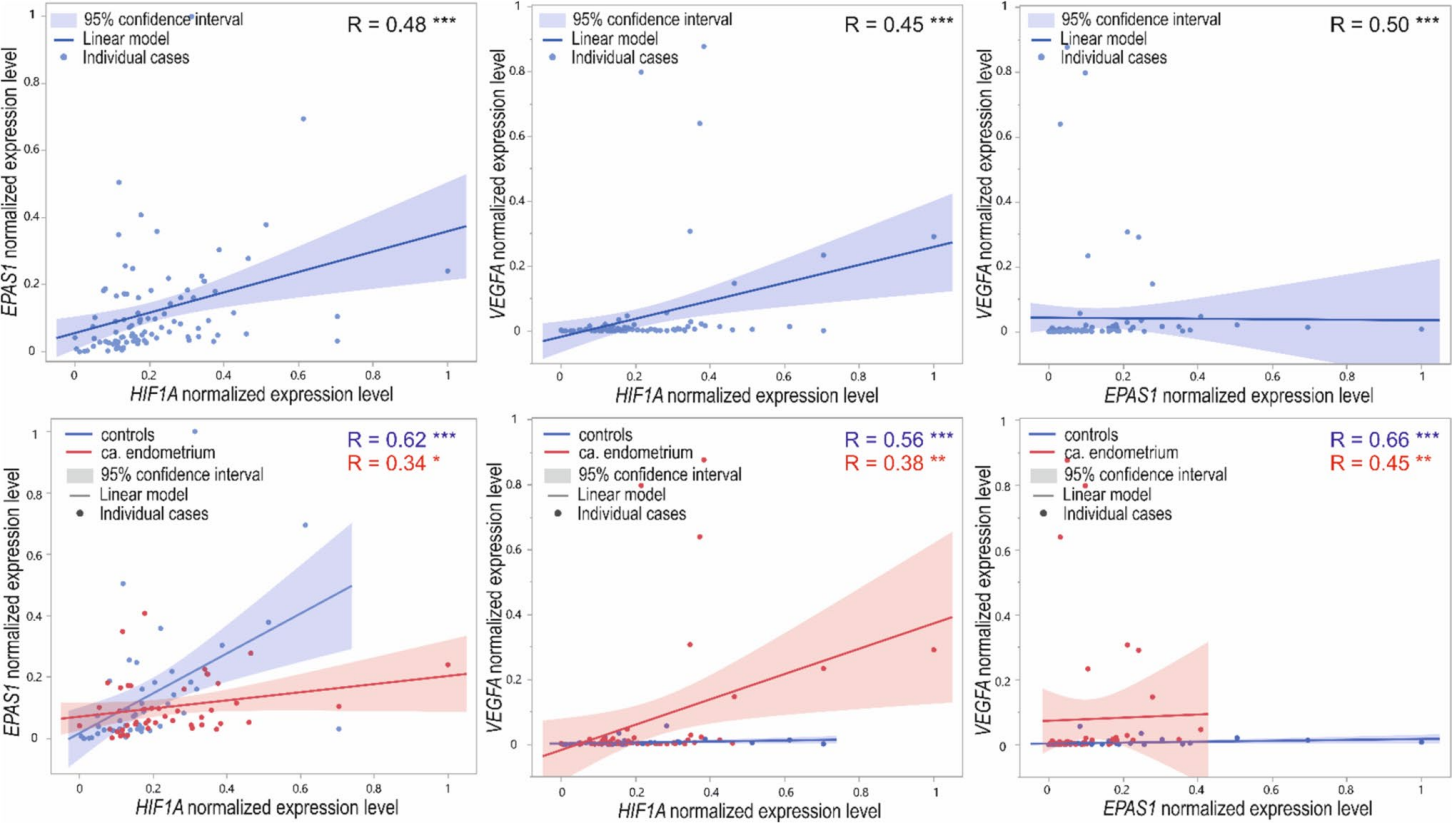
Dot-plot of normalized min–max expression level of *HIF1A*, *EPAS1*, and *VEGFA* normalized expression level in all cases (upper panel) and in controls and patients (lower panel); *p < 0.05, **p < 0.01, and ***p < 0.001; R, Spearman’s rank correlation coefficient

Moreover, we observed a positive, moderate, and statistically significant correlation between the expression level of all analyzed genes (*R* ranged from 0.45 to 0.5; *p* < 0.001) in the whole group of 92 cases (Fig. 5, upper panel). Next, in the EC subgroup, we observed a positive, weak, and statistically significant correlation between *HIF1A* and both *EPAS1* and *VEGFA* (*R* = 0.34; *p* = 0.02, and *R* = 0.38; *p* = 0.009, respectively). The expression of *EPAS1*, in turn, positively and moderately correlated with *VEGFA* (*R* = 0.45; *p* = 0.002)*.* In controls, the normalized expression level of all genes had positive and moderate correlations (*R* ranged from 0.56 to 0.66; *p* < 0.001; Fig. 5, lower panel).

**Fig. 6:**
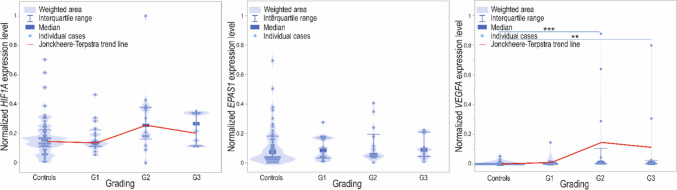
Violin-plot and significant Jonckheere-Tempestra trend (solid line) of *HIF1A*, *EPAS1*, and *VEGFA* normalized expression level controls and patients samples ordered by grading; **p < 0.01, ***p < 0.001

Analyzing the malignancy stage and GOIs, the expression level of *VEGFA* differed significantly (*p* = 0.0001) depending on the tumor grading (Fig. 6). The Dunn post-hoc tests with the Benjamini–Hochberg correction showed the difference between controls and tumor grade G2 (*p* = 0.0006) and G3 (*p* = 0.0076). We did not observe differences in the case of *HIF1A* and *EPAS1*. However, the Jonckheere-Terpstra test revealed a significant increasing trend of both *HIF1A* (*p* = 0.0357) and *VEGFA* (*p* < 0.0001) allied to increasing grading. A significant trend was not observed in the case of *EPAS1* (*p* > 0.05).

**Fig. 7:**
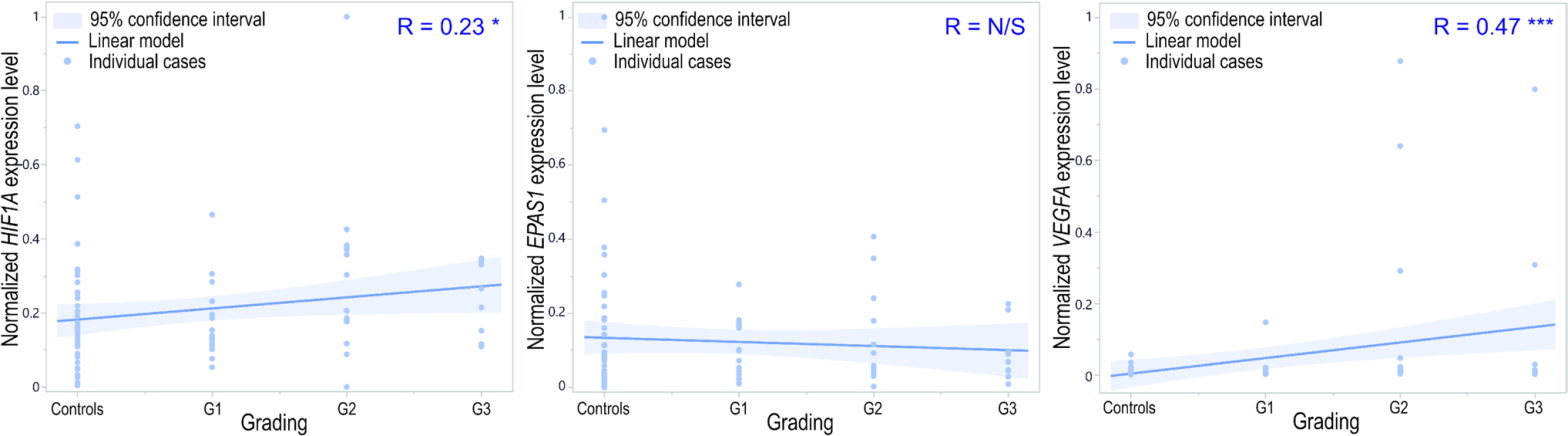
Dot-plot of normalized min–max expression level of *HIF1A*, *EPAS1*, and *VEGFA* normalized expression level with the increasing malignancy, from unchanged, control tissue to poorly differentiated—high grade G3 tumors; N/A, not available, N/S, not significant, *p < 0.05, ***p < 0.001; R, Spearman’s rank correlation coefficient

*HIF1A* expression levels positively, weakly, and significantly correlated with increasing malignancy, from unchanged control tissue to poorly differentiated, in high-grade G3 tumors (*R* = 0.23; *p* = 0.0289). Similarly, *VEGFA* expression levels moderately, positively, and significantly correlated with tumor grading (*R* = 0.47; *p* < 0.0001). The correlation between *EPAS1* and grading was insignificant (*p* > 0.05; Fig. 7).

### Menopausal status

**Fig. 8:**
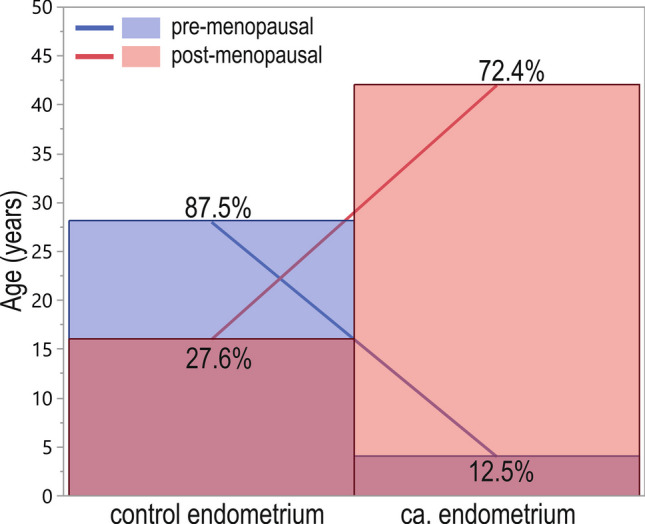
The pre-menopausal and post-menopausal ratio of cases in the control and cancer patients group

There was a significant difference in the number of cancer patients and controls regarding menopausal status (*p* < 0.001). We observed a higher percentage of cancer-manifested post-menopausal women (91%) compared to pre-menopausal women (9%). Only 12.5% of patients showed cancer presence before menopause, while the percentage was significantly higher (72.4%) after menopause. In the control group, 87.5% of women were pre-menopausal, and 27.6% were post-menopausal (Fig. 8). For pre-menopausal patients, the risk of cancer occurrence was decreased (odds ratio = 18.4 [95% CI 5.56–60.73]).

**Fig. 9:**
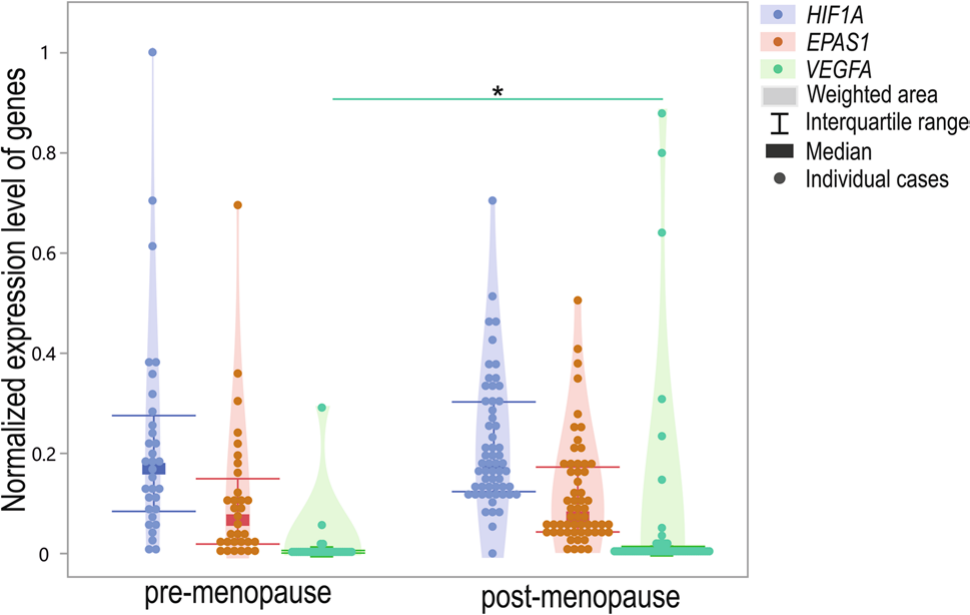
Violin-plot of *HIF1A*, *EPAS1*, and *VEGFA* normalized expression level in pre-menopausal and post-menopausal women; *p < 0.05

The expression level of *VEGFA* was significantly higher in post-menopausal compared to pre-menopausal patients (*p* = 0.0335). Neither *HIF1A* nor *EPAS1* differed between the mentioned groups (*p* > 0.05, Fig. 9).

**Fig. 10:**
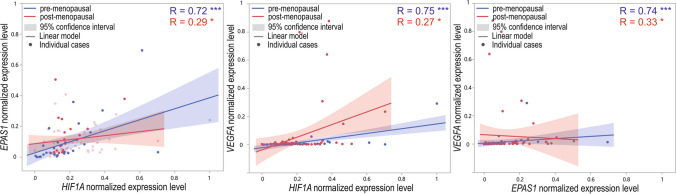
Dot-plot of normalized min–max expression level of *HIF1A*, *EPAS1*, and *VEGFA* normalized expression level in pre-menopausal and post-menopausal cases; *p < 0.05, ***p < 0.001; R, Spearman’s rank correlation coefficient

The expression of all analyzed genes had a significant and positive correlation. The correlation was strong in pre-menopausal cases, whereas it was weak in post-menopausal cases (Fig. 10). In tissues obtained from women before menopause, the correlation coefficient ranged from 0.72 to 0.75; *p* < 0.001. The weak correlation coefficient in post-menopausal cases ranged from 0.27 to 0.33 (*p* < 0.05).

**Fig. 11:**
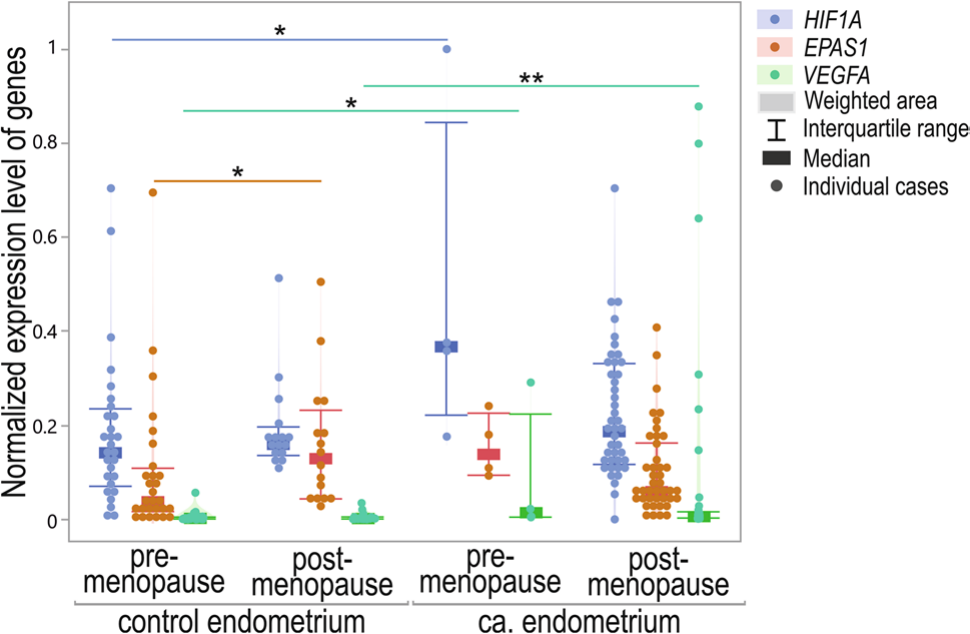
Violin-plot of *HIF1A*, *EPAS1*, and *VEGFA* normalized expression level in patients and control samples divided accordingly to menopausal status; *p < 0.05, **p<0.01

*HIF1A* and *VEGFA* gene expression (*p* = 0.0199 and *p* = 0.0239, respectively) differed significantly between the control and cancer groups in pre-menopausal women. Gene expression for both genes was lower in control samples. *EPAS1* expression did not differ between the analyzed cases (*p* = 0.0726). On the other hand, the expression of *VEGFA* was significantly higher in post-menopausal cancer patients (*p* = 0.0042). Additionally, the *EPAS1* level was higher in control endometrium tissues obtained from pre-menopausal women (*p* = 0.0228; Fig. 11).

**Fig. 12:**
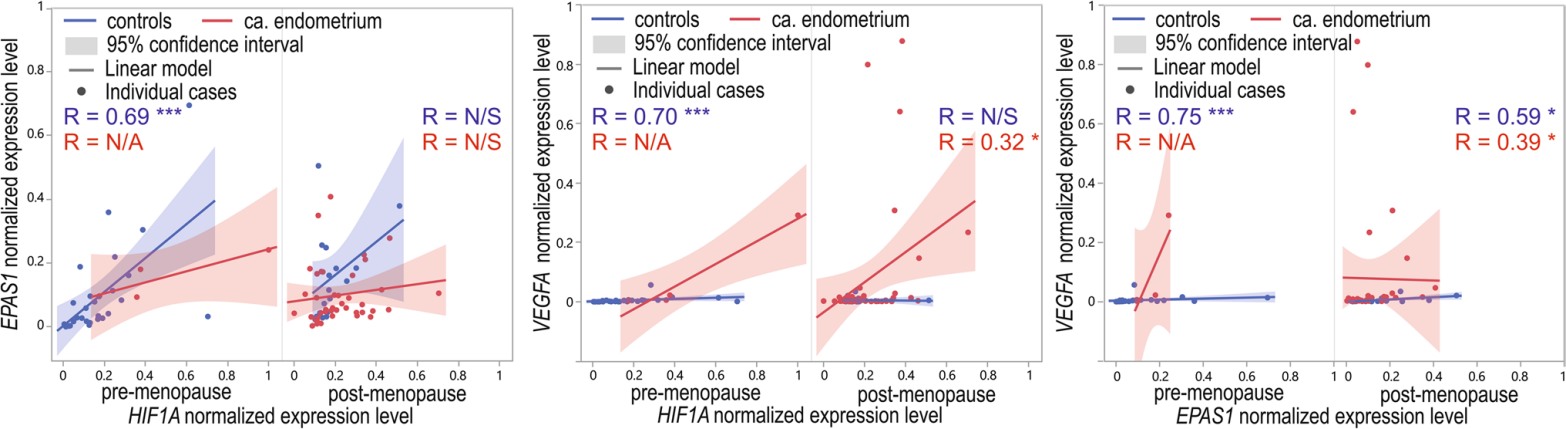
Dot-plot of normalized min–max expression level of *HIF1A*, *EPAS1*, and *VEGFA* normalized expression level in controls and EC tissue samples obtained from pre-menopausal and post-menopausal women; N/A, not available, N/S, not significant, *p < 0.05, ***p < 0.001; R, Spearman’s rank correlation coefficient

We observed a positive, strong, and significant (*p* < 0.001) correlation (correlations coefficients ranging from 0.69 to 0.75) between all analyzed genes in the control endometrium of non-menopausal patients (Fig. 12). *EPAS1* and *VEGFA* had a significant, moderate, and positive correlation in the control endometrium samples obtained from post-menopausal patients (*R* = 0.59; *p* = 0.0152, Fig. 12). In the tissue samples obtained from post-menopausal patients diagnosed with EC, *HIF1A* and *EPAS1* expression levels were significantly, weakly, and positively correlated with *VEGFA* (*R* = 0.32; *p* = 0.0411 and *R* = 0.039; *p* = 0.0102, respectively). We could not establish correlations’ coefficient values for post-menopausal cancer patients due to a limited number of cases (Fig. 12).

### Multivariable adjustment analyses

We carried out multiple linear regression analyses. The independent variable was the absence or presence of cancerous changes, and the dependent variables were age, BMI, comorbidity number, menopause status, and all analyzed gene expression levels. The best model of *R* = 0.66, *R*^2^ = 0.44 (adjusted *R*^2^ = 0.42; *p* < 0.0001) was shown in Table 2. Menopause status, BMI, and *HIF1A* expression were significant stimulating factors that influenced the cancer presence in patients compared to control groups. *EPAS1* gene expression was not significantly associated with the occurrence of changes, but it was close to the cut-off line (*p* = 0.0774). If the menopause status was removed from the regression model, age was considered instated as the factor of most significant influence, but the model was losing strength.

**Table 2:** Multiple logistic regression model for controls and cancer patients

Variables	*ß*	SE (*ß*)	*p* value
Menopause	0.55	0.08	** < 0.0001**
*HIF1A* Cr norm	0.26	0.09	**0.0037**
BMI	0.18	0.08	**0.0339**
*EPAS1* Cr norm^&^	− 0.16	0.09	0.0774
*VEGFA* Cr norm^&^﻿	0.08	0.10	0.3519
Age^&^	− 0.02	− 0.02	0.8654
Comorbidities number^&^	0.00	0.00	0.9752

^&^Variables not included in the model (not significant or redundant, due to menopause status in the model, the age was a redundant value); significant *p* values were indicated in bold

Due to the fact that age, BMI, and comorbidity number were related to cancer manifestation and could influence the expression level of analyzed genes, we used multivariable adjustment. The descriptive statistics for the adjusted group are shown in Table 3. *HIF1A* expression in the adjusted groups was higher in EC patients (*p* = 0.0006). Additionally, *VEGFA* expression was slightly, but not significantly, higher in controls (*p* = 0.0543). *EPAS1* did not differ between analyzed cases.

**Table 3:** *HIF1A*, *EPAS1*, and *VEGFA* expression in age, BMI, and comorbidities number adjusted group of analyzed cases

Variables	Control endometrium (*n* = 22)					Ca. endometrium (*n* = 24)					*p* values^‡^
	M	SD	Me	Q1	Q3	M	SD	Me	Q1	Q3	
Age	52	11.9	50	43	54	55	13.1	59	50	62	0.1728
BMI	27	2.8	27	26	28	30	7.0	29	24	34	0.0760
Comorbidities number	1	1	0	0	2	1	1	1	0	1	0.5851
*HIF1A* Cr norm	0.141	0.096	0.131	0.113	0.170	0.277	0.183	0.232	0.165	0.365	**0.0006**
*EPAS1* Cr norm	0.103	0.105	0.083	0.017	0.183	0.120	0.112	0.066	0.051	0.180	0.3972
*VEGFA* Cr norm	0.007	0.010	0.004	0.000	0.011	0.121	0.271	0.005	0.003	0.038	0.0543

*M*, mean; *SD*, standard deviation; *Me*, median; *Q1–Q3*, lower and upper quartile; ^‡^2-sided Mann–Whitney *U* test *p* value; significant *p* values were indicated in bold

## Discussion

EC represents one of the most frequent malignancies in females, and in 2020, it was the 14th most common cause of cancer-related mortality worldwide. An umbrella review conducted in 2018 revealed that obesity is the major risk factor for EC in both pre-menopausal and post-menopausal women. However, nulliparity, diabetes, and other hormonal and metabolic pathways involved in adiposity may also contribute to EC development (Raglan et al. 2019).

Moreover, recent studies emphasize that the effectiveness of EC therapy relies on the cellular and molecular mechanisms that interplay with the survival and resistance of tumor cells. During the tumor growth, the oxygen concentration and nutrient accessibility are decreased, which leads to hypoxia. Next, it activates angiogenesis-related and hypoxia-inducible factors, which are involved in the regulation of a vast number of genes. As a result, tumor cells must adapt to unfavorable conditions that may promote cancer metastasis and invasiveness (Salinas-Vera et al. 2022). Thus, we analyzed the *HIF1A*, *EPAS1*, and *VEGFA* gene expression patterns in cancerous and normal endometrial tissues involved in hypoxia, angiogenesis, and other biological processes and ultimately play a role in EC.

It is a dogma that numerous genes are involved in endometrial malignancies. The multiplicity of genes could illustrate the complexity of this issue, proteins, and other factors involved in the EPAS1, HIF1A, and VEGFA-related pathways (Supplementary Materials, Figure S1). It was shown that, e.g., *EPAS1* at the regulomics level has at least 31 upstream regulators and directly interacts with 27 proteins. Its expression is associated with more than ten cancer types, and its regulation on protein level is complex in physiological conditions and especially in malignancies (Kristan et al. 2021). The *HIF1A* regulation pathway is even more complex. Over 85 miRNAs have been reported to target this gene, and *HIF1A* downstream targets include protein-coding genes, long non-coding RNAs, and miRNAs (Kunej 2021).

**Fig. 13:**
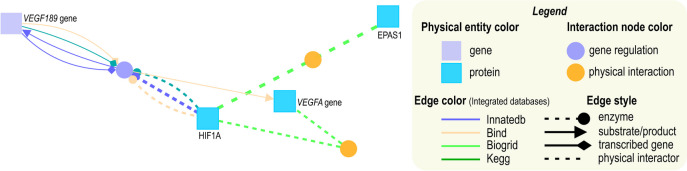
Map of the *HIF1A*, *EPAS1*, and *VEGFA* interplay (cpb.molgen.de)

In clinical practice, it would be almost impossible to search for diagnostic or prognostic candidate genes in the therapy of the future, so it is worth looking for only a few target genes. Thus, this work focused on angiogenesis, the hallmark of cancer growth, hypoxia, an angiogenesis driver, and three related to those processes genes (Fig. 13).

### Case–control study

The case–control study showed significant differences in age, BMI, and the number of comorbidities. These results, although not surprising, indicate that the group we studied is similar to the subpopulations analyzed by other authors (Nevadunsky et al. 2014). Moreover, as described by other authors, older women with EC were associated with more aggressive disease features, limited surgical or adjuvant therapy treatment, and a worse prognosis. Age could be a dependent or independent predictor of EC outcome. It was established that the higher age is related to a higher risk of disease recurrence and EC-specific mortality (Hag-Yahia et al. 2021; Biomorfologi et al. 2022). Age is not a modifiable factor, but aging affects the expression of genes. However, in our research, we did not study this issue.

Additionally, it was confirmed that obesity was associated with earlier EC occurrence or time of diagnosis (Nevadunsky et al. 2014; Gao et al. 2016; Park 2022). Metabolic and endocrine effects of obesity on EC susceptibility could be the explanation. Both higher BMI and age at diagnosis were observed to be strongly associated with EC (Nevadunsky et al. 2014). Primarily, obesity was confirmed to be positively related to the incidence of developing EC, particularly in perimenopausal women (Gao et al. 2016), but negatively in the early stage regardless of cancer subtypes and menopausal status (Gao et al. 2018). It is especially hazardous as patients are usually unaware of the obesity-related risk factor and BMI-related risk-reducing strategies. Women who lose weight have a better response to therapy and may also have improved oncological outcomes and result in fewer diagnosed comorbidities (Barr et al. 2021). Thus preventive for EC weight control should be taken as a standard approach (Park 2022). Patients should be informed and educated, e.g., by the physicians, and understand how comorbidities, lifestyle behavior, and modifiable factors affect their cancer risk.

As mentioned above, the comorbidity number was related to the cancer presence. The most common comorbidities were hypertension, obesity, diabetes, and cardiovascular diseases (Furau et al. 2021b, a). Multiple comorbidities, specifically those related to the metabolic syndrome, were also found to be more prevalent, e.g., in uterine cancer patients than in the general population, and this difference persisted after adjustment for age (Cook et al. 2013). Thus, we concluded, similarly to other authors, that medical programs should be undertaken toward treating comorbidities in EC to improve health quality and prolong survival and recurrence-free survival for these patients (Kurnit et al. 2015; Binder et al. 2016).

Primarily, we analyzed the molecular factors, such as *HIF1A*, *EPAS1*, and *VEGFA* gene expression levels, that could influence cancer risk and development. However, since the abovementioned age, BMI, and comorbidity number differed between cancer patients and the control group, we investigated if there is a relation and/or disturbances between those parameters and expression for the GOIs in these groups. Only *EPAS1* was weakly correlated with age in controls but not cancer patients. It seems that the analyzed GOIs expression levels are not affected by age, BMI, and comorbidities.

We analyzed the GOIs expression level and established differences between tissue specimens obtained from cancer-affected and control patients. *HIF1A* and *VEGFA* expression were significantly higher in cancerous tissue; *EPAS1* did not differ between those groups. Our results regarding *HIF1A* stay in line with the observations of other researchers. A meta-analysis of 25 studies showed positive associations of HIF1A protein overexpression with tumor grade, lymph node invasion, and invasion of the cancer-affected cells into the myometrium. It also seems to be a poor prognosis predictor in the EC (Zhu et al. 2020). In other gynecological malignancies—ovarian cancer, we also observed differences in *HIF1A* and *VEGFA* expression levels in a case–control study, but also *EPAS1* differed significantly (Englert-Golon et al. 2022). Similarly to the results shown in this study, the *VEGFA* expression was higher in cancer-affected tissue, whereas the HIF1A protein was at a lower level in malignant ovarian tumors. Thus mutual relationships in the expression of both *HIF1A* and *EPAS1* and *VEGFA* but also with other genes and proteins could be of importance also in endometrial cancer progression (Englert-Golon et al. 2022). As *HIF1A* plays an essential role in the adaptive cellular response to hypoxia and is associated with poor clinical outcomes, it seems to be a promising therapeutic target in the EC course of treatment. Thus, several selective and non-selective inhibitors directly or indirectly target the *HIF1A* upstream or downstream signaling pathway and, as a result, decrease this protein level (Seeber et al. 2010).

Moreover, previous studies showed that the modulation of hypoxia-inducible factors and vascular endothelial growth factor A might contribute to the activation of downstream signaling pathways involved in cancer progression and related processes such as neovascularization (D’amico et al. 2021). As a result, hypoxia-induced angiogenesis triggers the epithelial-mesenchymal transition process (Maugeri et al. 2016, 2021). Additionally, mutual relationships of both *HIF1A* and *EPAS1* with *VEGFA* play an important role in the oncogenesis and progression of other gynecological malignancies (Englert-Golon et al. 2022). Over the past years, the proteins HIF1A (Semenza 2000; Melillo 2006), EPAS1 (Petrella and Brinckerhoff 2009; Wigerup et al. 2016; Singhal et al. 2019), and VEGFA (Terme et al. 2013; English et al. 2017; Yang et al. 2018) have been shown to be eligible targets for anti-tumor therapies.

We observed significantly higher expression levels of *VEGFA* in endometrial epithelial tumors (Dziobek et al. 2019). *VEGFA* was shown to be regulated together with *HIF1A* by dipeptidyl peptidase IV, which plays a direct role in the progression of several human malignancies (Khin et al. 2003; Beckenkamp et al. 2015; Yang et al. 2017). VEGFA, with VEGFB and their receptors, are crucial proteins involved in the development of new blood vessels. If the blood flow is suboptimal, its expression depends on a hypoxia environment (Dziobek et al. 2019). We observed stronger relations between the gene-to-gene expression level in tissues that lack cancerous changes (correlation coefficients 0.56–0.66) compared to neoplasia-affected tissues (correlation coefficients 0.34–0.45). Similar observations were made in the case of ovarian malignancies, where in non-affected tissue, the expression levels of all analyzed genes were significantly and more strongly correlated than in benign changes and malignant tumors (Englert-Golon et al. 2022). It could imply that mutual relations of the analyzed genes or disturbances in their expression control are either a cause or a reason for EC manifestation. However, it was shown that comparing HIF1A to EPAS1 proteins, despite their primary role as transcription factors for cellular response to hypoxia, could play independent or/and coregulatory roles in tumor physiology and progression (Davis et al. 2022; Song et al. 2022).

*VEGFA* showed the highest dependence of all analyzed genes with tumor grading. We established significant differences between control and tumor grades G2 and G3 and a significant increasing trend in the growth of the *VEGFA* expression level with the grade. The same tendency was shown in ovarian tumors, and *VEGFA* expression intensified with the pathological process progress (Englert-Golon et al. 2022). Similarly to *VEGFA*, regarding *HIF1A* in endometrial tissue*,* a significant trend was observed with increasing grading but not in the case of *EPAS1*. However, we did not observe differences in the expression levels for either *HIF1A* or *EPAS1*. We also observed a significant correlation between both *HIF1A* and *VEGFA* expression levels and tumor grading but not in the case of *EPAS1*. It was estimated that HIF1A proteins positively correlated with poor prognosis in EC (Seeber et al. 2010), and it was also shown that VEGFA factors significantly differed between tumor grading (Dziobek et al. 2019). Both are fundamental proteins involved in the hypoxia-vascularization axis and properly nourish and oxygenate non-pathological and cancer cells (El-Sayed Mohammed Youssef et al. 2015; Dziobek et al. 2019). The increase in HIF1A and VEGFA factors was shown to be significantly, directly, and positively associated with increased malignancy in other types of cancer, e.g., oral squamous cell carcinoma (El-Sayed Mohammed Youssef et al. 2015; Mahecha and Wang 2017). Thus, similarly to *HIF1A*, at least the *VEGFA* gene could be a promising target for anticancer therapy or promote the efficacy of different treatments (Song et al. 2022).

### Menopausal status and GOIs expression

The additional analyzed parameter was the menopausal status of the patients. Among analyzed GOIs, only the *VEGFA* expression level was significantly higher in tissue obtained from post-menopausal women compared to pre-menopausal women. However, in the case of gene-to-gene correlations, stronger associations were observed in pre-menopausal women (correlation coefficients 0.72–0.75 vs. 0.27–0.33, respectively). However, it could also be a combined effect of the menopausal and cancer-presence-related status described above. At the same time, we observed a significantly higher percentage of cancer manifested in women after menopause. We suspect, and it could not be excluded, that the effect of menopausal status and cancer manifestation on GOIs expression is mutual in the case of these patients. Thus, we also examined the gene expression differences in case–control studies between pre- and post-menopausal cases. *HIF1A* and *VEGFA* expression levels in pre-menopausal controls were lower than in pre-menopausal EC cases. In cancerous tissue samples obtained from post-menopausal subjects, the *VEGFA* level was higher compared to controls. In turn, the *EPAS1* level was higher in non-affected controls before menopause.

Interestingly, in non-cancer-affected controls, all gene-to-gene correlations were positive and strong in pre-menopausal cases and were either moderate for *VEGFA* and *EPAS1* or not significant for other gene-to-gene correlations in post-menopausal. In cancer-affected tissue, significant correlations of *VEGFA* with *HIF1A* and *EPAS1* were weak and only in post-menopausal cases. These results suggest that in cancer cases and especially after menopause, the mutual relations between analyzed genes weaken and pass over control mechanisms disturbing angiogenesis and the hypoxia balance. As a result, it explains that this process leads to tissue abnormalities. There is still not a very clear understanding of the association between age, menopause, and EC (Wu et al. 2019). Therefore, it is important to analyze other genetic and epigenetic factors that could be allied with cancer susceptibility and progression. Our investigation follows the need to examine the modifiable and non-modifiable factors related to the patient’s condition and the association of *HIF1A*, *EPAS1*, and *VEGFA* with EC risk and progression.

### Multivariable adjustment analyses

We conducted multiple linear regression analyses as many factors could influence cancer manifestation. The most significant contributing factors to EC were menopause status, increasing *HIF1A* levels, and a high BMI. Age was excluded as a redundant factor to the menopause value. Interestingly, *EPAS1* expression was the first under the cut-off line and, therefore, would not exclude this gene as being of minor importance in the susceptibility to cancer.

Additionally, we adjusted the analyzed cases to age, BMI, and comorbidity number using multivariable adjustments. After adjustments, the controls and cancer-affected tissue samples showed significantly higher *HIF1A* expression and slightly, but not significantly, higher *VEGFA* expression levels. The multivariate analysis showed that classical risk factors are not the only reason for cancer manifestation in patients. To better understand EC biology, we should consider factors leading to changes in gene expression, especially angiogenesis and hypoxia-related genes. The clinical effectiveness of chemotherapy is variable, suggesting that novel molecular targeted therapies against pathways associated with cancer cell survival in EC treatment are needed. As a result, they may impair the cellular processes activated by hypoxia in the tumor microenvironment (Salinas-Vera et al. 2022).

### Limitation of the study

This study also has potential limitations. The groups’ characteristics, in terms of age, BMI, and comorbidities number, differ significantly before adjustment. This issue may influence the presented results, which should be interpreted cautiously. The results are promising, and in-depth analyses, also at protein level in larger sample sizes may validate our observations. Still, as compared to other studies cancer types, and also, e.g., murine models, the analyzed by us genes could deal even as independent prognostic factors and could be potential target for anti-tumor therapies (Joshi et al. 2014; Beuselinck et al. 2018; Wierzbicki et al. 2019; Englert-Golon et al. 2022). Although in this work, we evaluated the mRNA expression of analyzed genes. We are aware that the tissue-specific protein effective translation could differ at some point. However, the effective translation of mRNA to protein was proven in many papers, not only in human endometrial cancer cells (Downes et al. 2018), in endometrial unchanged and cancer-affected specimens (Sivridis et al. 2002; Maybin et al. 2018), but also in other tissues (Maugeri et al. 2016, 2021; Wierzbicki et al. 2019). Additionally, the Human Protein Atlas database showed that most tissues exhibited moderate to strong VEGFA cytoplasmatic staining, weak to moderate HIF1A cytoplasmic and/or nuclear immunoreactivity (The Human Protein Atlas. https://www.proteinatlas.org; Accessed 21 Oct 2022), and other sources confirmed effective translation of *EPAS1* gene (Sivridis et al. 2002).

## Conclusions

As reported, the classic factors such as age, weight, comorbidity number, and menopausal status are inseparable from the rising rate of cancer presence. We should also consider angiogenesis and hypoxia-related genes as a cause or outcome of cancer changes. Additionally, the gene-to-gene relation could, in the same way, be seen as either a diagnostic or therapeutic target in EC. The physicians should first inform the patients about the modifiable risk factors such as BMI. Second, more attention should be paid to diagnosing patients with comorbidities in older age and after menopause. Our results suggest that in cancer cases, especially after menopause, the mutual relations between analyzed genes weaken and pass over control mechanisms that disturb angiogenesis and the hypoxia balance. As a result, it explains that this process leads to tissue abnormalities. There is still not a very clear understanding of the association between age, menopause, and EC. The phenomenon is more complex due to constant physiological changes in endometrial tissue, e.g., menstruation, natural aging processes, and/or normal response to hormonal fluctuations. Therefore, the hypoxia-related genes serve as an angiogenic master switch. These factors should be considered in designing angiogenesis and hypoxia-related gene-targeting therapies.

## Supplementary Information

Below is the link to the electronic supplementary material.

Supplementary file1 (PDF 4882 KB)

Supplementary file2 (DOCX 18 KB)

## Data Availability

The datasets used and analyzed during the current study are available from the corresponding author on reasonable request.
