# Supplementary material for: HIF1A, EPAS1, and VEGFA: angiogenesis and hypoxia-related gene expression in endometrium and endometrial epithelial tumors
Source: J Appl Genet. 2025 Jan 31;67(1):139–53. doi: 10.1007/s13353-025-00939-7 (PMC12819458; doi:10.1007/s13353-025-00939-7)
Supplement: Supplementary file 1 — Supplementary file1 (PDF 4882 KB) [file 13353_2025_939_MOESM1_ESM.pdf]

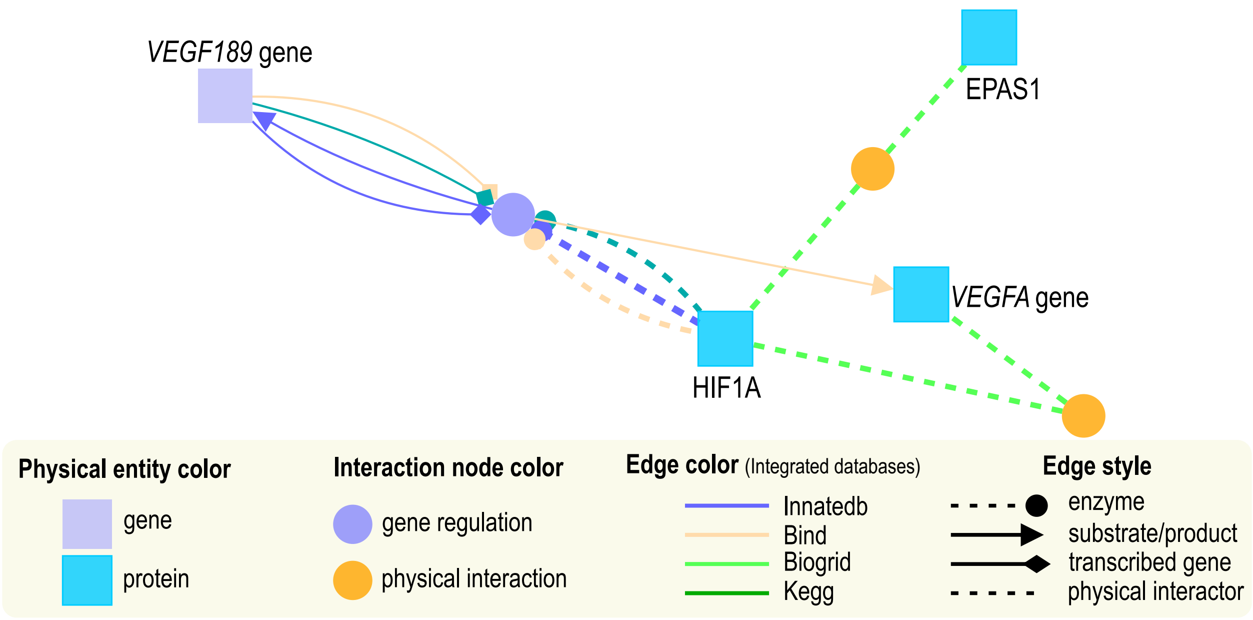

VEGF189 gene
EPAS1
VEGFA gene
HIF1A
Physical entity color
gene
protein
Interaction node color
gene regulation
physical interaction
Edge color (Integrated databases)
Innatedb
Bind
Biogrid
Kegg
Edge style
enzyme
substrate/product
transcribed gene
physical interactor

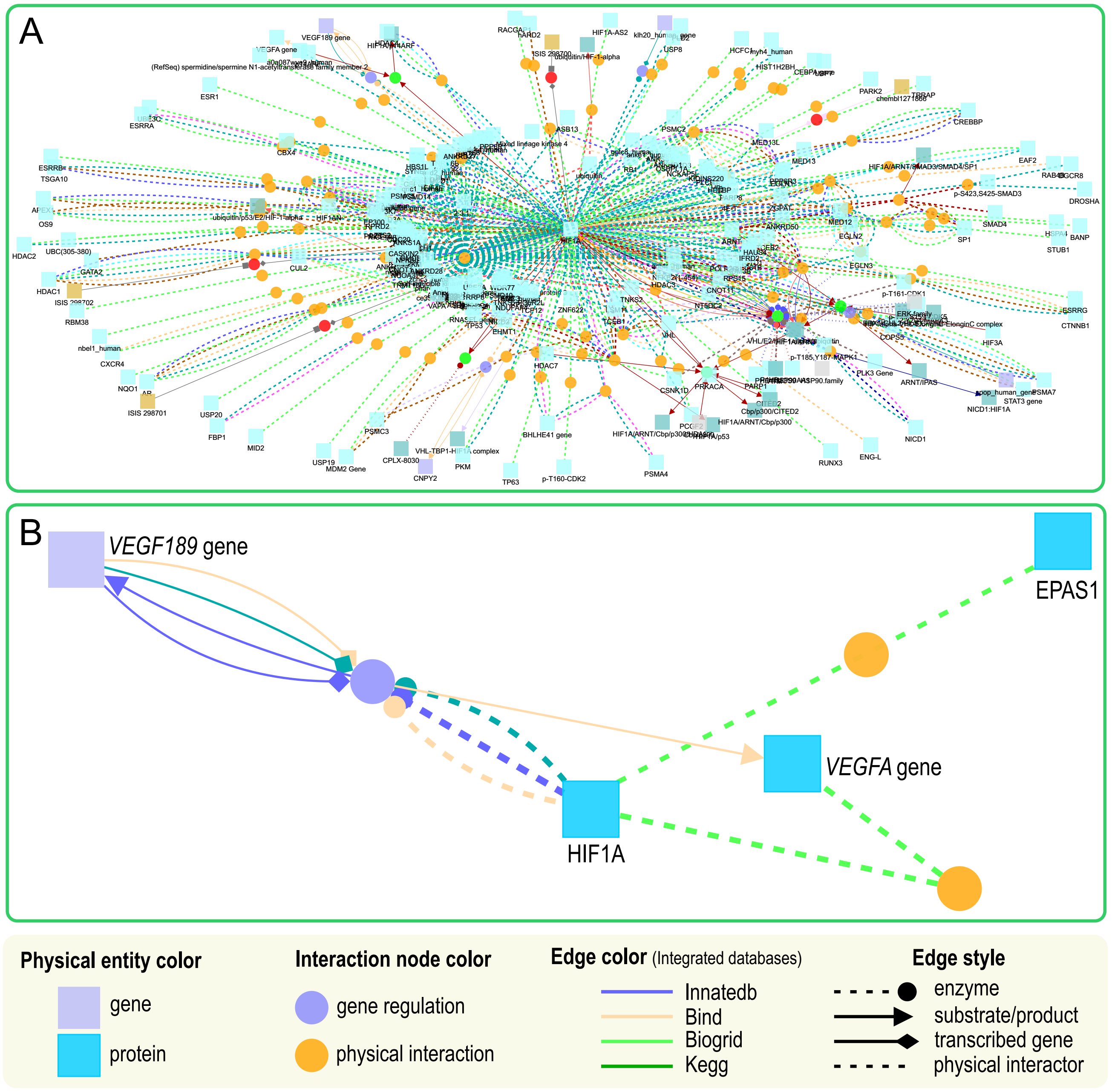
A
B
VEGF189 gene
EPAS1
VEGFA gene
HIF1A
Physical entity color
gene
protein
Interaction node color
gene regulation
physical interaction
Edge color (Integrated databases)
Innatedb
Bind
Biogrid
Kegg
Edge style
enzyme
substrate/product
transcribed gene
physical interactor

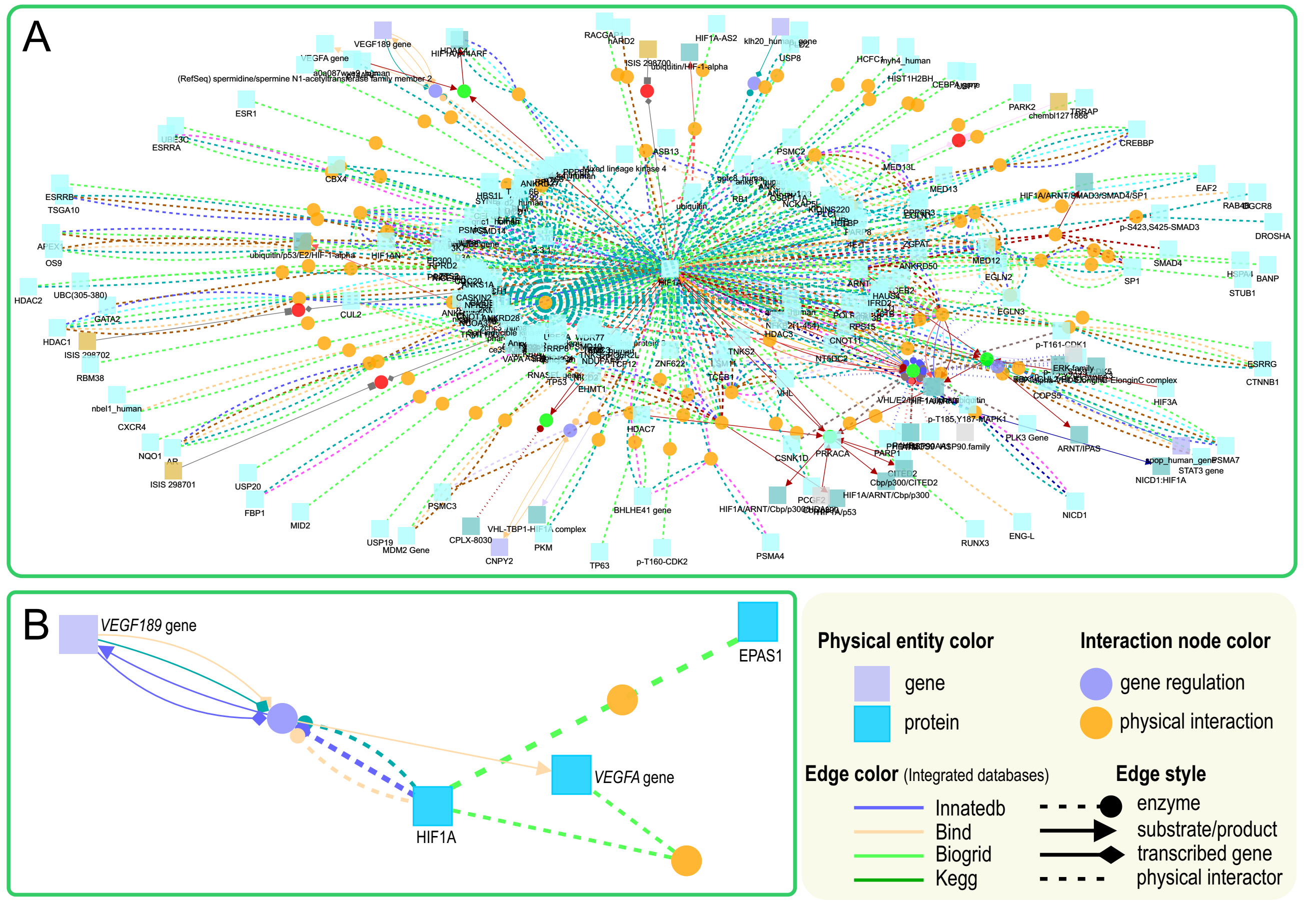

A
B
VEGF189 gene
EPAS1
VEGFA gene
HIF1A
Physical entity color
gene
protein
Interaction node color
gene regulation
physical interaction
Edge color (Integrated databases)
Innatedb
Bind
Biogrid
Kegg
Edge style
enzyme
substrate/product
transcribed gene
physical interactor

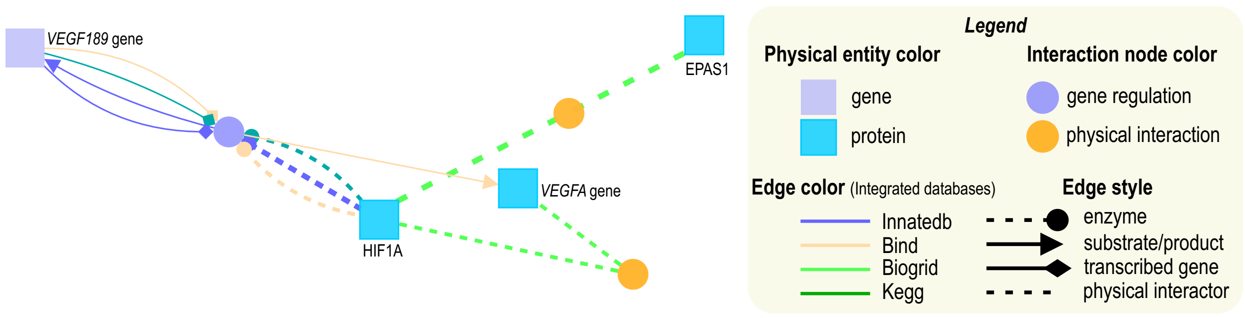
VEGF189 gene
EPAS1
VEGFA gene
HIF1A
Legend
Physical entity color
gene
protein
Interaction node color
gene regulation
physical interaction
Edge color (Integrated databases)
Innatedb
Bind
Biogrid
Kegg
Edge style
enzyme
substrate/product
transcribed gene
physical interactor

Supplementary materials

Journal of Applied Genetics

HIF1A, *EPAS1*, and *VEGFA*: Angiogenesis and Hypoxia-Related Gene Expression in Endometrium and Endometrial Epithelial Tumors

Monika Englert-Golon[1] (ORCID: 0000-0003-1110-8693), Małgorzata Tokłowicz[2,†] (0000-0003-0091-6492), Aleksandra Żbikowska[2,†] (ORCID: 0000-0002-6891-8569), Stefan Sajdak[1] (0000-0002-8641-4188), Małgorzata Kotwicka[2] (ORCID: 0000-0002-9802-374X), Paweł Jagodziński[3] (ORCID: 0000-0002-9046-6802), Andrzej Pławski[4] (ORCID: 0000-0002-0534-8941), Mirosław Andrusiewicz[2,*] (ORCID: 0000-0002-8781-3447)

[1] Department of Gynecology, Division of Gynecologic Oncology, Poznan University of Medical Sciences, Polna 33 St., 60-535 Poznań, Poland; mgolon@ump.edu.pl (M.E-G.)

[2] Chair and Department of Cell Biology, Faculty of Health Sciences, Poznan University of Medical Sciences, Rokietnicka 5D, 60-806 Poznań, Poland; mtoklowicz@ump.edu.pl (M.T.), azbikowska@ump.edu.pl (A.Ż.), mkotwic@ump.edu.pl (M.K.), andrus@ump.edu.pl (M.A.).

[3] Department of Biochemistry and Molecular Biology, Poznan University of Medical Sciences, Święcickiego 6 St., 61-701 Poznań, Poland; pjagodzi@ump.edu.pl (P.J.)

[4] Institute of Human Genetics, Polish Academy of Sciences, Strzeszyńska 32 St., 60-479 Poznań, Poland; andrzej.plawski@igcz.poznan.pl (A.P.)

*Correspondence: andrus@ump.edu.pl

[†] These authors contributed equally to this work.

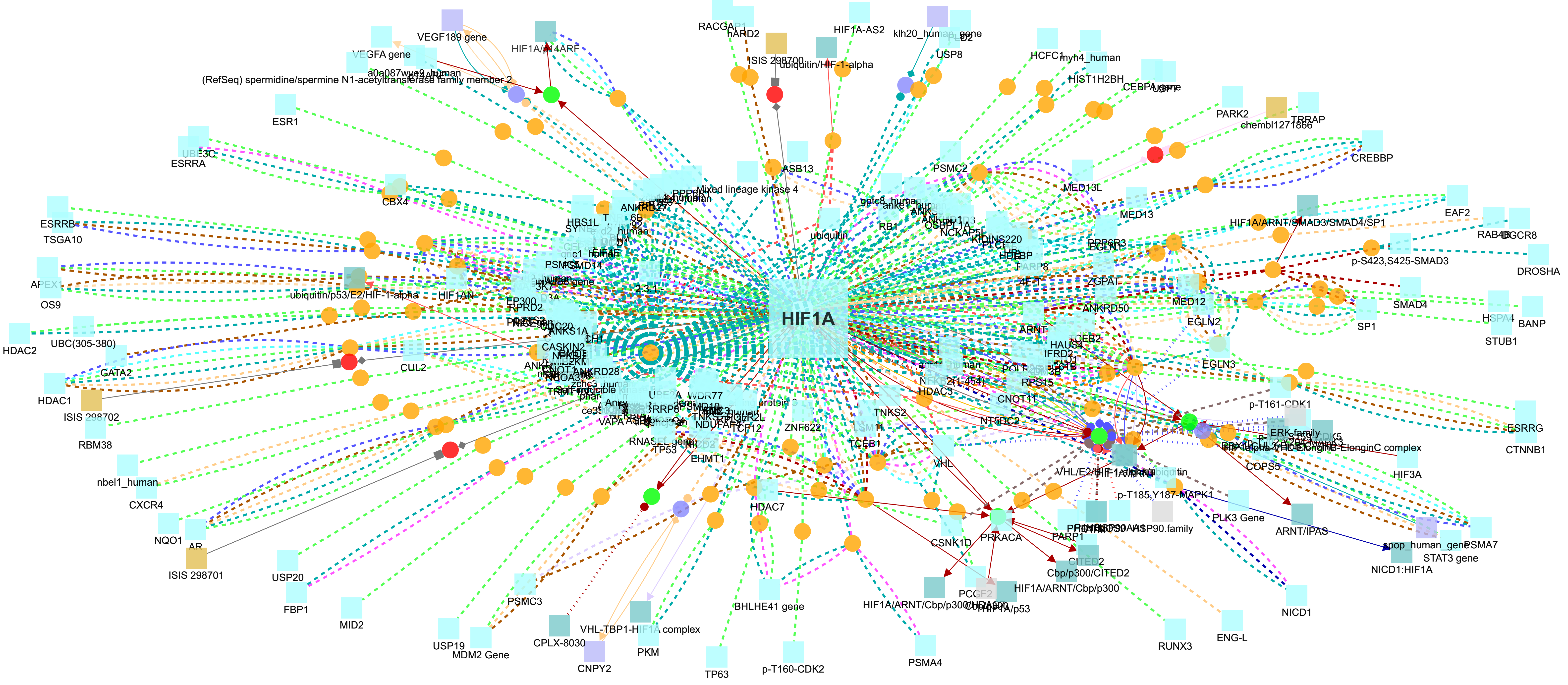

Figure S1. Map of the HIF1A, EPAS1, and VEGFA interactions. Panel A show one of the possible different proteins and genes interactions model, with the HIF1A as a central point, combining different signal paths.
