# Supplementary material for: HIF1A, EPAS1, and VEGFA: angiogenesis and hypoxia-related gene expression in endometrium and endometrial epithelial tumors
Source: J Appl Genet. 2025 Jan 31;67(1):139–53. doi: 10.1007/s13353-025-00939-7 (PMC12819458; doi:10.1007/s13353-025-00939-7)
Supplement: Supplementary file 2 — Supplementary file2 (DOCX 18 KB) [file 13353_2025_939_MOESM2_ESM.docx]

Supplementary materials

*Journal of Applied Genetics*

*HIF1A*, *EPAS1,* and *VEGFA*: Angiogenesis and Hypoxia-Related Gene Expression in Endometrium and Endometrial Epithelial Tumors

Monika Englert-Golon^1^ (ORCID: 0000-0003-1110-8693), Małgorzata Tokłowicz^2,†^ (0000-0003-0091-6492), Aleksandra Żbikowska^2,†^ (ORCID: 0000-0002-6891-8569), Stefan Sajdak^1^ (0000-0002-8641-4188), Małgorzata Kotwicka^2^ (ORCID: 0000-0002-9802-374X), Paweł Jagodziński^3^ (ORCID: 0000-0002-9046-6802), Andrzej Pławski^4^ (ORCID: 0000-0002-0534-8941), Mirosław Andrusiewicz^2,*^ (ORCID: 0000-0002-8781-3447)

^1^ Department of Gynecology, Division of Gynecologic Oncology, Poznan University of Medical Sciences, Polna 33 St., 60-535 Poznań, Poland; mgolon@ump.edu.pl (M.E-G.), ssajdak@ump.edu.pl (S.S.)

^2^ Chair and Department of Cell Biology, Faculty of Health Sciences, Poznan University of Medical Sciences, Rokietnicka 5D, 60-806 Poznań, Poland; mtoklowicz@ump.edu.pl (M.T.), azbikowska@ump.edu.pl (A.Ż.), mkotwic@ump.edu.pl (M.K.), andrus@ump.edu.pl (M.A.).

^3^Department of Biochemistry and Molecular Biology, Poznan University of Medical Sciences, Święcickiego 6 St., 61-701 Poznań, Poland; pjagodzi@ump.edu.pl (P.J.)

^4^Institute of Human Genetics, Polish Academy of Sciences, Strzeszyńska 32 St., 60-479 Poznań, Poland; andrzej.plawski@igcz.poznan.pl (A.P.)

***** Correspondence: andrus@ump.edu.pl

^†^ These authors contributed equally to this work.

Table S1. Spearman’s rank correlation coefficients of analyzed genes with age, BMI and comorbidities number.

|  | All cases | | | Controls | | | Ca. endometrium | | |
| --- | --- | --- | --- | --- | --- | --- | --- | --- | --- |
|  | N | R | *p*-value | N | R | *p*-value | N | R | *p*-value |
| **Age** | | | | | | | | | |
| *HIF1A* Cr norm | 90 | .07 | .5064 | 44 | .22 | .1536 | 46 | -.18 | .2442 |
| *EPAS1* Cr norm | 90 | .21 | **.0492** | 44 | .33 | **.0269** | 46 | .11 | .4735 |
| *VEGFA* Cr norm | 90 | .25 | **.0161** | 44 | .17 | .2643 | 46 | .08 | .6063 |
| **BMI** | | | | | | | | | |
| *HIF1A* Cr norm | 89 | .00 | .9944 | 43 | .04 | .8231 | 46 | -.09 | .5414 |
| *EPAS1* Cr norm | 89 | -.05 | .6460 | 43 | .02 | .9053 | 46 | -.13 | .3920 |
| *VEGFA* Cr norm | 89 | .22 | **.0389** | 43 | .03 | .8238 | 46 | .19 | .1948 |
| **Comorbilities number** | | | | | | | | | |
| *HIF1A* Cr norm | 89 | .12 | .2579 | 43 | .14 | .3729 | 46 | .01 | .9226 |
| *EPAS1* Cr norm | 89 | -.03 | .7799 | 43 | .13 | .3963 | 46 | -.25 | .0975 |
| *VEGFA* Cr norm | 89 | .17 | .1198 | 43 | .20 | .1954 | 46 | -.06 | .6840 |

**Legend:** N – number of cases; R – Spearman’s rank correlation coefficient; BMI – body mass index; significant correlations’ coefficients are in bold
